# Sociodemographic and patient reported outcomes by racial and ethnicity status among participants in a randomized controlled trial for methamphetamine use disorder

**DOI:** 10.1016/j.dadr.2024.100230

**Published:** 2024-04-06

**Authors:** Chukwuemeka N. Okafor, Thomas Carmody, Angela L. Stotts, Gavin Bart, Taryn L. Mayes, Tara Karns-Wright, Madhukar Trivedi, Steve Shoptaw, Jennifer S. Potter

**Affiliations:** a Department of Medicine, Division of Infectious Diseases, Long School of Medicine, University of Texas Health Science Center San Antonio, San Antonio, TX, USA; b Peter O’Donnell Jr. School of Public Health, University of Texas Southwestern Medical Center, Dallas TX, USA; c Center for Depression Research and Clinical Care, Peter O’Donnell Jr. Brain Institute and Department of Psychiatry, University of Texas Southwestern Medical Center, Dallas, TX, USA; d McGovern Medical School, University of Texas Health Science Center at Houston, TX, USA; e University of Minnesota Medical School, Minneapolis, USA; f Department of Psychiatry and Behavioral Sciences, University of Texas Health Science Center San Antonio, San Antonio, TX, USA; g Department of Family Medicine, University of California Los Angeles, Los Angeles, CA, USA; h Department of Psychiatry and Biobehavioral Sciences, University of California Los Angeles, Los Angeles, CA, USA

**Keywords:** Methamphetamine use disorder, Treatment, Racial and ethnic disparities, Patient reported outcomes

## Abstract

**Background:**

There has been a significant increase in methamphetamine use and methamphetamine use disorder (Meth UD) in the United States, with evolving racial and ethnic differences.

**Objectives:**

This secondary analysis explored racial and ethnic differences in baseline sociodemographic and clinical characteristics as well as treatment effects on a measure of substance use recovery, depression symptoms, and methamphetamine craving among participants in a pharmacotherapy trial for Meth UD.

**Methods:**

The ADAPT-2 trial (ClinicalTrials.gov number, NCT03078075; N=403; 69% male) was a multisite, 12-week randomized, double-blind, trial that employed a two-stage sequential parallel design to evaluate the efficacy of combination naltrexone (NTX) and oral bupropion (BUP) vs. placebo for Meth UD. Treatment effect was calculated as the *weighted mean change* in outcomes in the NTX-BUP minus placebo group across the two stages of treatment.

**Results:**

Of the 403 participants in the ADAPT-2 trial, the majority (65%) reported non-Hispanic White, while 14%, 11% and 10% reported Hispanic, non-Hispanic Black, and non-Hispanic other racial and ethnic categories respectively. At baseline non-Hispanic Black participants reported less severe indicators of methamphetamine use than non-Hispanic White. Treatment effects for recovery, depression symptoms and methamphetamine cravings did not significantly differ by race and ethnicity.

**Conclusions:**

Although we found racial and ethnic differences at baseline, our findings did not show racial and ethnic differences in treatment effects of NTX-BUP on recovery, depression symptoms and methamphetamine cravings. However, our findings also highlight the need to expand representation of racial and ethnic minority groups in future trials.

## Highlights

• Participants received NTX and BUP (vs. placebo) for methamphetamine use disorder.
• Non-Hispanic Black participants reported less severe indicators of methamphetamine use at baseline.
• Non-Hispanic Black participants were more likely to be male and to report reported living with HIV.
• Treatment effects for depression, craving and recovery were similar across racial and ethnic groups.
• Study was limited by the modest sample size for racial and ethnic minorities.

## 1 Introduction

Between 2015 and 2019, the number of persons reporting past-year methamphetamine use and DSM-IV methamphetamine use disorder (Meth UD) increased by 43% (from 1.4 million to 1.6million), and 62% (from 675,000–1.09 million) respectively (Han et al., 2021a). Drug overdose death rates involving psychostimulants such as methamphetamine surged by 317% between 2013 and 2019 (Mattson et al., 2021). Notably, there are racial and ethnic differences in the methamphetamine use epidemic and drug overdose death rates involving psychostimulants in the United States. For instance, Black individuals experienced a tenfold increase in the prevalence of past-year Meth UD without injection, the highest among all racial and ethnic groups (Han et al., 2021a). Additionally, Black patients in urban areas had the highest odds of injecting methamphetamine in 2019 (Pro et al., 2022), a method associated with worse treatment outcomes for Meth UD (McKetin et al., 2018). Moreover, drug overdose deaths involving methamphetamine rose fastest among Black men from 2011 to 2018 (Han et al., 2021b), while Hispanic individuals saw faster increases in such deaths compared to non-Hispanic White individuals in various regions of the U.S (Townsend et al., 2022). Factors such as economic disparities, higher rates of poverty, unemployment, and limited access to healthcare in Black and Hispanic communities, along with systemic racism, contribute to these disparities in methamphetamine use (Amaro et al., 2021, Farahmand et al., 2020).

No FDA- approved medications exists for the treatment of Meth UD. Medication combinations have been studied for the treatment of Meth UD with limited efficacy (Chan et al., 2019, Chan et al., 2020) with one recent exception. The Accelerated Development of Additive Pharmacotherapy Treatment (ADAPT-2) trial, a combination of intramuscular naltrexone and oral bupropion produced a higher treatment response (defined as when compared to placebo (Trivedi et al., 2021). However, few studies have assessed racial and ethnic differences in patient-reported outcomes associated with methamphetamine use.

The goal of this analysis is to examine: (1) Whether there are baseline racial and ethnic differences in sociodemographic characteristics (e.g., employment status), and clinical (HIV, psychiatric disorders) status, and (2) Whether there are racial and ethnic differences in treatment effects for secondary patient-reported outcomes (including global functioning, depression symptoms, and methamphetamine cravings) among participants in the ADAPT-2 trial. We hypothesized that non-Hispanic Black and Hispanic individuals would show more severe clinical and psychosocial characteristics at baseline compared to non-Hispanic White individuals. We further hypothesized that non-Hispanic Black and Hispanic individuals will have less favorable patient-reported outcomes than non-Hispanic White individuals.

## 2 Materials and methods

### 2.1 Study design and procedures

This analysis utilized data from the Accelerated Development of Additive Treatment for Methamphetamine Disorder (ADAPT-2). Details of the ADAPT-2 study has been previously described (Trivedi et al., 2021). Briefly, ADAPT-2 was a multisite, 12-week randomized, double-blind, trial evaluating the safety and efficacy of extended-release injectable naltrexone (380 mg/3 weeks) combined with once-daily oral extended-release bupropion (450 mg/day) compared to matching placebo [i.e., NTX-BUP vs. placebo]. The trial employed a sequential parallel comparison design (SPCD) (Fava et al., 2003) in which study participants were randomized in a 1:3 ratio during the first 6-weeks to receive NTX-BUP or placebo (Stage 1). In the second 6-weeks (Stage 2) of follow-up, participants in the placebo group who did not have a response (defined as at least three methamphetamine-negative urine tests out of a possible four at the end of the first 6-weeks period) were rerandomized in a 1:1 ratio (Stage 2).

### 2.2 Participants

Participants (N=403) were adults aged 18–65 years who met DSM-5 criteria for moderate to severe Meth UD (Hasin et al., 2013), who wanted to quit or reduce methamphetamine use, reported methamphetamine use on at least 18 of the 30 days before consent, had 2+ methamphetamine-positive urine samples obtained at least 2 days apart within 10 days before randomization; and were opioid free at the time of randomization. Participants undergoing treatment for substance use disorder, expecting a need for opioid-containing medications during the trial (e.g., planned surgery) were excluded. Participants were classified based on self-reported race and ethnicity as non-Hispanic Black, non-Hispanic White, non-Hispanic Other, and Hispanic.

## 3 Measures

### 3.1 Treatment recovery

The treatment effectiveness assessment (TEA) was used to assess treatment progress and recovery, based on participants perspectives in four domains of functioning: health, lifestyle, community, and substance use (Ling et al., 2012, Ling et al., 2019). Scores for each domain range from 1 to 10, and total scores across all four domains range from 4 to 40, with higher scores indicating improved (or *better* if at baseline) functioning. The TEA has demonstrated acceptable internal consistency and construct validity in the current sample (Vo et al., 2023).

### 3.2 Depression symptoms

The 9-item patient health questionnaire (PHQ-9) was used to assess severity of depression symptoms across the last two weeks. Total summed score across the 9-items ranging from 0 to 27, with higher scores indicating greater depression symptom severity (Kroenke et al., 2001). The PHQ-9 has demonstrated acceptable internal consistency in individuals with substance use disorders (Bentley et al., 2021, Dum et al., 2008).

### 3.3 Methamphetamine craving

The study used a Visual Analogue Scale (VAS) to assess methamphetamine craving over the past week (McHugh et al., 2014), with the VAS ranging from 0 (no craving) to 100 (most intense craving possible).

### 3.4 Statistical analysis

We used frequencies, percentages (for categorical variables), medians or means (for continuous variables) to describe the sample overall and by race and ethnicity status at baseline. Chi-square tests (for categorical variables) and one-way analysis of variance or Wilcoxon tests were used to compare baseline sociodemographic, clinical, and psychosocial characteristics by non-Hispanic White compared to the non-Hispanic other race and ethnicity groups. Reported p-values have been Bonferroni corrected for 3 comparisons. The overall treatment effect *h* for this sequential parallel comparison design was calculated as the *weighted mean change* of the patient reported outcomes in the NTX-BUP group in Stage 1 and Stage 2 minus the *weighted mean change* in the placebo group in Stage 1 and Stage 2, using the pre-specified weight (0.43 for Stage 1 and 0.57 for Stage 2). Treatment effect *h* for each of the patient reported outcomes was calculated separately for each race and ethnicity group (within-group treatment effect *h*) using a repeated-measures mixed effects model for a continuous outcome (Doros et al., 2013). The model contained covariates for the baseline value of each patient reported outcome measure, age, sex, baseline days of methamphetamine use and baseline days of tobacco use. Reported p-values were Bonferroni corrected for 4 comparisons. PHQ-9 and methamphetamine craving were assessed weekly while the TEA (recovery) was assessed only at baseline, Week 6, and Week 12. Comparison of treatment effect between White non-Hispanic and the other race and ethnicity groups was made by t-test with Bonferroni correction for 3 comparisons. We selected non-Hispanic White individuals as the reference group in between group comparisons, because we were interested the difference between historically marginalized racial and ethnic groups versus non-Hispanic White individuals.

## 4 Results

### 4.1 Baseline sample description

Majority of the sample (mean age=41 years, SD=10) were male (69%), completed some college degree (65%), and non-Hispanic White (64%). Altogether, 11% of the sample were non-Hispanic Black, 14% were Hispanic and 10% self-reported non-Hispanic other race.

### 4.2 Baseline differences by racial and ethnicity status

All racial and ethnic groups reported significantly shorter duration (in years) of methamphetamine use compared to non-Hispanic White participants (all p’s <0.04) (Table 1). Non-Hispanic Black participants reported older age (in years) at initiation of methamphetamine (30 vs. 24; p<0.001). Non-Hispanic Black participants had a higher proportion of self-reporting being male (94% vs. 62%; p<0.001) and living with HIV (39% vs. 17%; p=.002), compared to non-Hispanic White participants. Non-Hispanic Black participants also had higher proportions self-reporting being never married (74% vs. 44%) than non-Hispanic White participants (Table 1). Hispanic participants were younger (41 vs. 36; p=0.003) and had a higher proportion of self-reporting sex with men and women among males (78% vs. 54%; p=0.018) when compared to non-Hispanic White counterparts (Table 1).

**Table 1:** Baseline characteristics of participants in the trial by race/ethnicity status.

Characteristics	All, N=403	Non-Hispanic Black, N=46	Non-Hispanic White, N=260	Non-Hispanic Other**, N=42	Hispanic, N=55
Age, mean (SD) π	40.76 (10.1)	42.80 (10.9)	41.38 (9.9)	40.33(11.0)	36.44 (8.9)
Male, n (%) ‡	277 (68.7)	43 (93.5)	162 (62.3)	29 (69.0)	43 (78.1)
Sexual orientation behavior π					
MSM/W	151 (61.4)	25 (67.6)	78 (54.2)	16 (66.7)	32 (78.1)
MSW	95 (38.6)	12 (32.4)	66 (45.8)	8 (33.3)	9 (21.9)
Employment, n (%) †					
Working	156 (38.7)	11 (23.9)	103 (39.6)	14 (33.3)	28 (50.9)
Unemployed	150 (39.7)	20 (43.4)	111 (42.6)	12 (28.5)	17 (30.9)
Other	87 (21.5)	15 (31.6)	46 (17.6)	16 (38.1)	10 (18.1)
Marital Status, n (%) ‡					
Married/cohabiting	93 (23.1)	8 (17.4)	64 (24.6)	11 (26.2)	10 (18.2)
Never married	204 (50.6)	34 (73.9)	115 (44.2)	23 (54.8)	32 (58.2)
Separated/Divorced/Widowed	104 (25.8)	4 (8.7)	79 (30.4)	8 (19.1)	13 (23.6)
Education, n (%)					
Less than High School	35 (8.8)	3 (6.5)	22 (8.5)	5 (11.9)	5 (9.1)
High School or GED	107 (26.6)	12 (26.1)	70 (26.9)	8 (19.0)	17 (30.9)
Some college	261 (64.7)	31 (67.3)	168 (64.6)	29 (69.0)	33 (60.0)
HIV+ Status, n (%) ‡	90 (22.3)	18 (39.1)	44 (16.9)	12 (28.5)	16 (29.0)
Days tobacco use in past month π	30 (0, 30)	28.5 (0, 30)	30 (0.5, 30)	30 (14, 30)	28 (0, 30)
Psychiatric disorder, n (%)					
Anxiety	207 (51.4)	20 (43.5)	133 (51.2)	26 (61.9)	28 (50.9)
Major Depression	177 (43.9)	20 (43.5)	118 (45.4)	19 (45.2)	20 (36.4)
Eating Disorder	16 (3.9)	1 (2.1)	10 (3.8)	2 (4.7)	3 (5.4)
ADHD	106 (26.3)	8 (17.3)	74 (28.4)	8 (19.0)	16 (29.0)
Bipolar Disorder	57 (14.1)	5 (10.8)	37 (14.2)	9 (21.4)	6 (10.9)
Schizophrenia	12 (2.9)	3 (6.5)	7 (2.6)	1 (2.3)	1 (1.8)
Methamphetamine use					
Days used in past month, mean (SD)	26.6 (4.1)	25.9 (4.4)	26.9 (3.9)	26.1 (4.4)	26.3 (4.2)
Age of first Use, mean (SD)‡	24.8 (9.9)	30.2 (11.5)	23.7 (8.8)	26.8 (12.0)	23.9 (10.0)
Duration of Use, mean (SD)‡ π †	15.9 (10.4)	12.5 (8.8)	17.6 (10.5)	13.5 (11.2)	12.5 (8.8)
Most frequent route of use, n (%)					
Nasal or oral	33 (8.19)	3 (6.52)	24 (9.23)	2 (4.76)	4 (7.27)
Smoking	293 (72.7)	37 (80.4)	181 (69.6)	33 (78.6)	42 (76.4)
IV injection	77 (19.1)	6 (13.0)	55 (21.2)	7 (16.7)	9 (16.4)
Methamphetamine Craving, mean (SD)‡	66.1 (22.3)	56.2 (25.9)	67.6 (21.3)	65.2 (23.3)	67.8 (21.1)
TEA, Total score mean (SD)‡	18.29 (7.2)	21.28 (7.3)	17.90 (7.0)	19.38 (6.9)	17.02 (7.7)
PHQ-9, mean (SD)	10.88 (6.5)	10.20 (6.3)	10.92 (6.5)	10.74 (6.8)	11.38 (6.4)

**Note:** ‡ *P*<0.05 for Black vs. Non-Hispanic White; † *P*<0.05 for Other vs. Non-Hispanic White; π *P*<0.05 for Hispanic vs. Non-Hispanic White; *P*-values are based on a 1-way ANOVA, or Wilcoxon test (for continuous variables) or Chi-square tests (for categorical variables); ║Includes retired/student; **Comprised of American Indian or Alaska Native, n=13 and Asian, n=14 and Native Hawaiian or Pacific Islander and some other race (n=15). Numbers (and percents) do not always add to the total due to missing values from nonresponse.

### 4.3 Baseline racial and ethnic differences in patient reported outcomes

Non-Hispanic Black participants reported statistically significant lower methamphetamine craving (56 vs. 68; p=0.004; in VAS on a scale of 0–100) at baseline compared to non-Hispanic White participants. Baseline total recovery scores from the TEA were statistically significantly higher in non-Hispanic Black participants compared to non-Hispanic White participants (21 vs. 18; p=0.009).

### 4.4 Racial and ethnicity differences in treatment effect on patient reported outcomes

There was a significant treatment effect for depression symptoms among Hispanics only (*h*=-2.8, p=0.046). Treatment effects on recovery scores from the TEA was also significant within non-Hispanic Whites (*h*=3.1, p=0.004), Hispanics (*h*=7.8, p=0.004) and non-Hispanic other race (*h*=8.0, p=0.002; Table 2). In adjusted analysis comparing treatment effects by race/ethnicity status (when designated non-Hispanic Whites as the reference group), we did not find statistically significant differences in treatment effects for either patient reported outcomes (Table 2). There was a significant treatment effect on methamphetamine craving for non-Hispanic Blacks (*h=*-13.5, p=0.042) and non-Hispanic Whites only (*h=* −9.3, p<0.001). In adjusted analysis comparing treatment effects by race/ethnicity status (when designated non-Hispanic Whites as the reference group), we did not find statistically significant differences in treatment effects (Table 2).

**Table 2:** Treatment effect *h* of NTX-BUP vs. Placebo within and between race/ethnicity status.

	**PHQ-9**			**TEA Total score**			**MA Craving**		
**Racial and ethnic Subgroups**	***h*****(95% CI)**	**P-value‡**	**P-value†**	***h*****(95% CI)**	**P-value‡**	**P-value†**	***h*****(95% CI)**	**P-value‡**	**P-value†**
Non-Hispanic White	-0.8 (1.9, 0.4)	0.77	Ref	3.1 (0.9, 5.3)	0.01	Ref	-9.3 (-14.7, −3.7)	<0.01	Ref
Non-Hispanic Black	-0.7 (-3.8, 2.4)	0.99	0.99	0.4 (-4.1, 4.9)	0.99	0.84	-13.5 (-26.5, −0.6)	0.16	0.99
Hispanic	-2.8 (-5.5, −0.1)	0.18	0.56	7.8 (2.5, 13.1)	0.01	0.32	-7.7 (-17.9, 2.4)	0.54	0.99
Non-Hispanic Other	-2.9 (-5.8, 0.0)	0.22	0.53	7.9 (2.8, 13.1)	0.00	0.25	-13.0 (-28.1, 2.1)	0.36	0.99

**Note: ‡**P-values are for mean change from baseline in treatment effect *h within* racial and ethnic subgroups and were adjusted for 4 multiple comparisons using the Bonferroni correction; **†**P-values are for comparisons in treatment effect *h between* racial and ethnic subgroups and were adjusted for 3 multiple comparisons using the Bonferroni correction

## 5 Discussion

In this secondary analysis of participants in the ADAPT-2 trial of NTX-BUP (vs placebo) for Meth UD, we found no statistically significant differences in treatment effects on methamphetamine craving, depression symptoms and recovery *between* racial and ethnic groups. Few studies have explicitly compared treatment outcomes by race and ethnicity among persons receiving treatment for methamphetamine use disorder. However, our finding is consistent with studies showing comparable treatment outcomes among racial and ethnic groups in treatment studies for psychostimulants (including cocaine dependence) (Jordan et al., 2022, Montgomery and Carroll, 2017, Miguel et al., 2019). The lack of significant treatment effect between race and ethnicity groups might reflect the modest sample sizes in the racial and ethnic minority groups. Future methamphetamine trials should enroll larger samples of racial and ethnic minorities to examine potential differences in depression and craving symptoms.

In the current analysis, we also found that at entry into the ADAPT-2 trial, non-Hispanic Black participants reported less severe methamphetamine use characteristics including older age at methamphetamine onset, shorter years of methamphetamine use and lower methamphetamine cravings ratings (VAS) than most racial and ethnic groups, but particularly when compared to non-Hispanic Whites. This study's findings are consistent with previous epidemiological research and observations from drug treatment programs, which indicate that non-Hispanic Black individuals generally begin using stimulants, such as methamphetamine, at an older age compared to non-Hispanic Whites (McCabe et al., 2007, Sj et al., 2009, Deutsch-Link et al., 2023), which could explain the overall less severe methamphetamine use characteristics in non-Hispanic participants in this study. This pattern may be attributed to factors such as social and cultural norms, as well as historically limited access to and preference for methamphetamine within Black communities (Copes et al., 2014). Although further research is needed to better understand the factors contributing to racial and ethnic differences in methamphetamine use characteristics, appropriate treatment approaches that consider age at initiation and other methamphetamine use characteristics are necessary.

Regarding baseline sociodemographic differences, non-Hispanic Black participants were more likely to be male and to report living with HIV when compared to non-Hispanic White participants. In addition, among males, Hispanic participants had a higher proportion reporting sex with men and women compared to non-Hispanic whites. There are established associations between methamphetamine use and poor treatment outcomes for persons with HIV (Fulcher et al., 2021, Goodman-Meza et al., 2019) and HIV acquisition (Okafor et al., 2020, Plankey et al., 2007). It is possible that outreach efforts to address methamphetamine use, may have encouraged male non-Hispanic Black participants living with HIV and Hispanics men who sex with men and women to seek treatment options in the ADAPT-2 trial. Moreover, this finding underscores the importance for clinicians to recognize and address the intersectionality of identities, such as race/ethnicity, gender, and HIV status, when assessing and treating individuals with Meth UD.

The current study is among the very few studies that have examined baseline differences in clinical and psychosocial characteristics by race and ethnicity, and secondary treatment outcomes among participants in a RCT for methamphetamine use disorder. However, a few limitations of this study are worth noting. Our study was a secondary analysis of the ADAPT-2 trial which was designed to assess the efficacy of a combination medication for methamphetamine use disorder and as such was limited in the measures collected from participants. In addition, the sample sizes were modest for racial and ethnic minority groups and there were low numbers of females particularly among non-Hispanic Blacks and Hispanic participants. Therefore, the findings reported in this study may have not be generalizable to individuals from racial and ethnic minority groups, including women from these groups.

In conclusion, in this secondary analysis of participants in the ADAPT-2 trial, we found no statistically significant differences in treatment effects on methamphetamine craving, depression symptoms and recovery *between* racial and ethnic groups. However, non-Hispanic Black participants appear to have less severe indicators of methamphetamine use and are more likely to report being male and living with HIV. Because of the modest sample sizes of the racial and ethnic minority groups future trials of methamphetamine use disorder should increase the representation of racial and ethnic minority groups, including women from these groups. This would facilitate examining the intersectionality of identities such as race, ethnicity, gender to help unique challenges and inform tailored interventions.

## Funding

Dr. Okafor is supported by the National Institutes on Drug Abuse (grant# K01DA047918).

## CRediT authorship contribution statement

**Steve Shoptaw:** Writing – review & editing. **Jennifer S Potter:** Writing – review & editing. **Tara Karns-Wright:** Writing – review & editing. **Madhukar Trivedi:** Writing – review & editing. **Gavin Bart:** Writing – review & editing. **Taryn L Mayes:** Writing – review & editing. **Angela L Stotts:** Writing – review & editing. **Chukwuemeka N Okafor:** Conceptualization, Writing – original draft, Writing – review & editing. **Thomas Carmody:** Formal analysis.

## Declaration of Competing Interest

Authors declare none.
